# Supplementary material for: Cytokinin-mediated trichome initiation in Nicotiana benthamiana following Agrobacterium tumefaciens infiltration
Source: Plant Commun. 2026 Jun 17;7(10):101971. doi: 10.1016/j.xplc.2026.101971 (PMC13681608; doi:10.1016/j.xplc.2026.101971)
Supplement: Supplementary file 1 [file mmc1.pdf]

Plant Communications, Volume *7*

Supplemental information

Cytokinin-mediated trichome initiation in *Nicotiana benthamiana* following *Agrobacterium tumefaciens* infiltration

Alejandro Brand, Robert Säbel, Dario Esposto, Gerd U. Balcke, Frank Syrowatka, Stephanie Krüger, Claudia Horn, Sylvestre Marillonnet, and Alain Tissier

Supplementary Figures and Materials and Methods

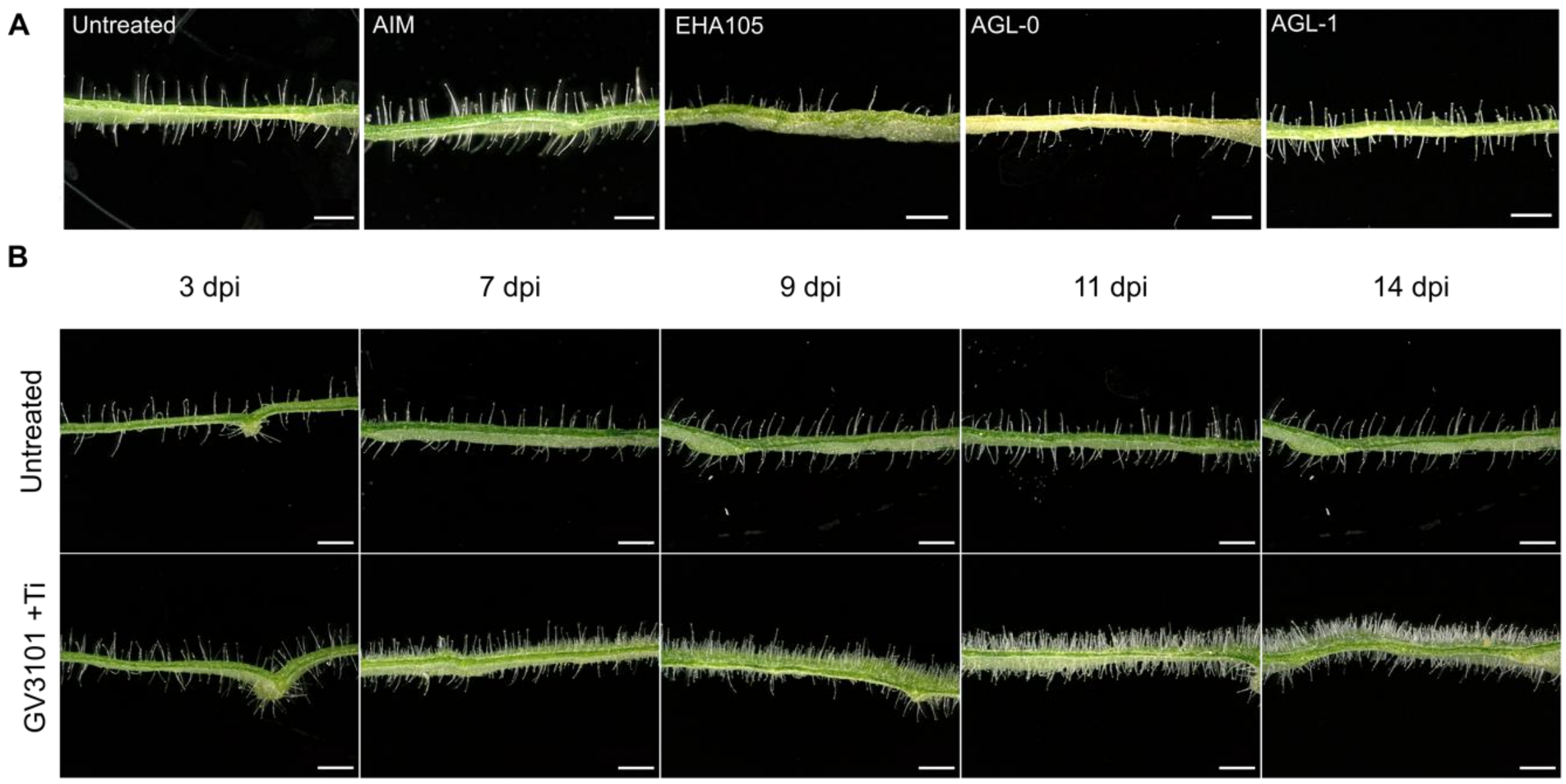

Supplementary Figure 1. (A) Trichome development on the adaxial and abaxial sides of leaves of *Nicotiana benthamiana* infiltrated by different *Agrobacterium tumefaciens* strains 14 days post-infiltration (dpi). AIM: Agrobacterium infiltration medium. **(B)** Trichome density over time after infiltration with GV3101 + Ti. Scale bar = 1 mm. Ti, refers to pMP90.

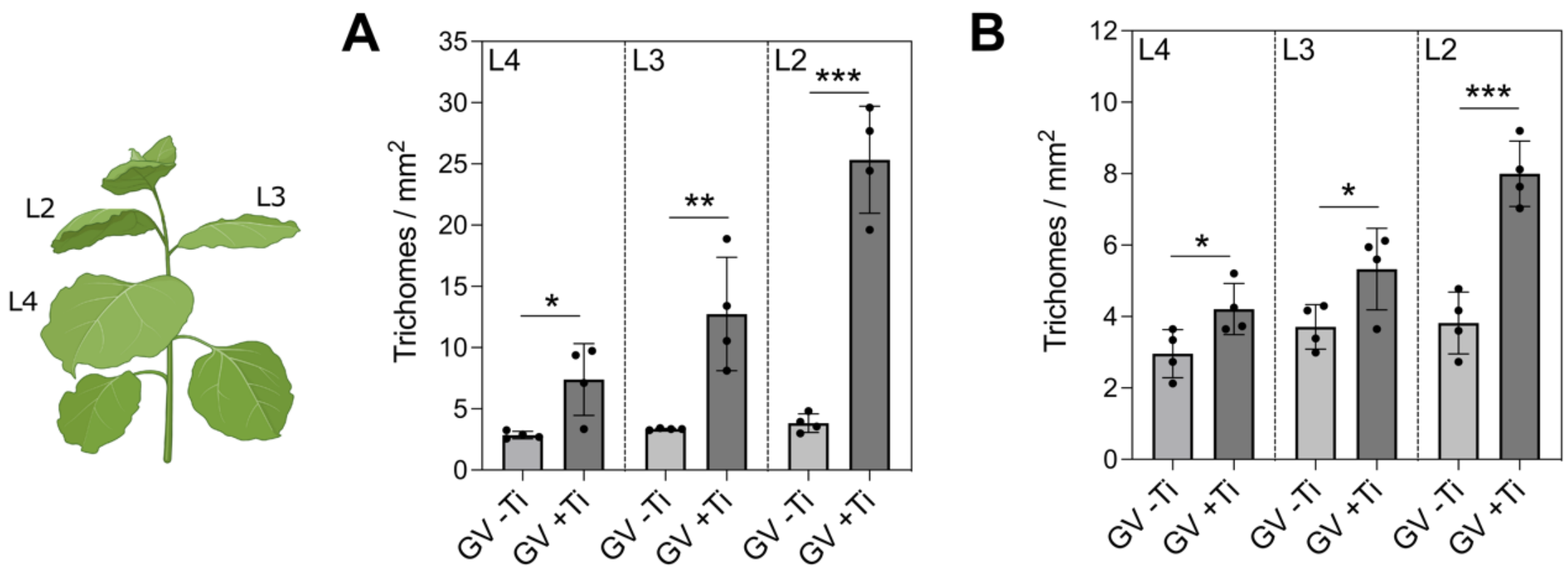

Supplementary Figure 2. Trichome counting using leaves of three developmental stages. Trichomes were counted on the adaxial **(A)** and abaxial **(B)** side of the leaves after 12 dpi (*n* = 4 biological replicates). Pairwise comparisons were conducted using Student's *t*-test (*$p < 0.05$, **$p < 0.01$, ***$p < 0.001$). Error bars represent standard deviation. GV: GV3101; Ti: pMP90. Created in BioRender. Brand Duran, A. (2026) https://BioRender.com/pd207hz.

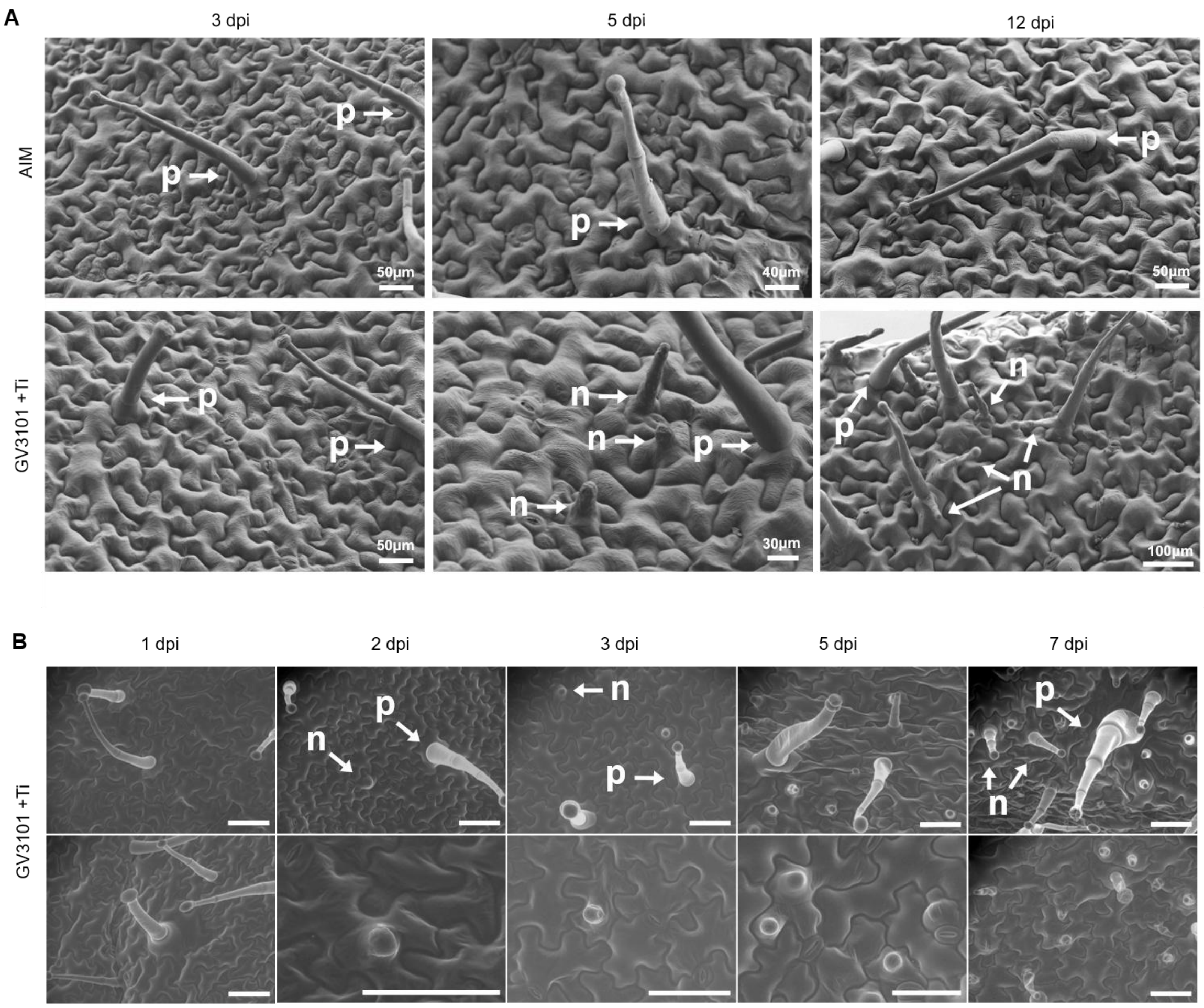

Supplementary Figure 3. (A) Scanning electron microscopy (SEM) and **(B)** Environmental SEM of trichome initiation after *Agrobacterium* infiltration. Mock AIM: Agrobacterium infiltration medium; Ti: pMP90 Ti plasmid; p: preexisting trichomes; n: newly emerging trichomes. Scale bar in B: 100 µm.

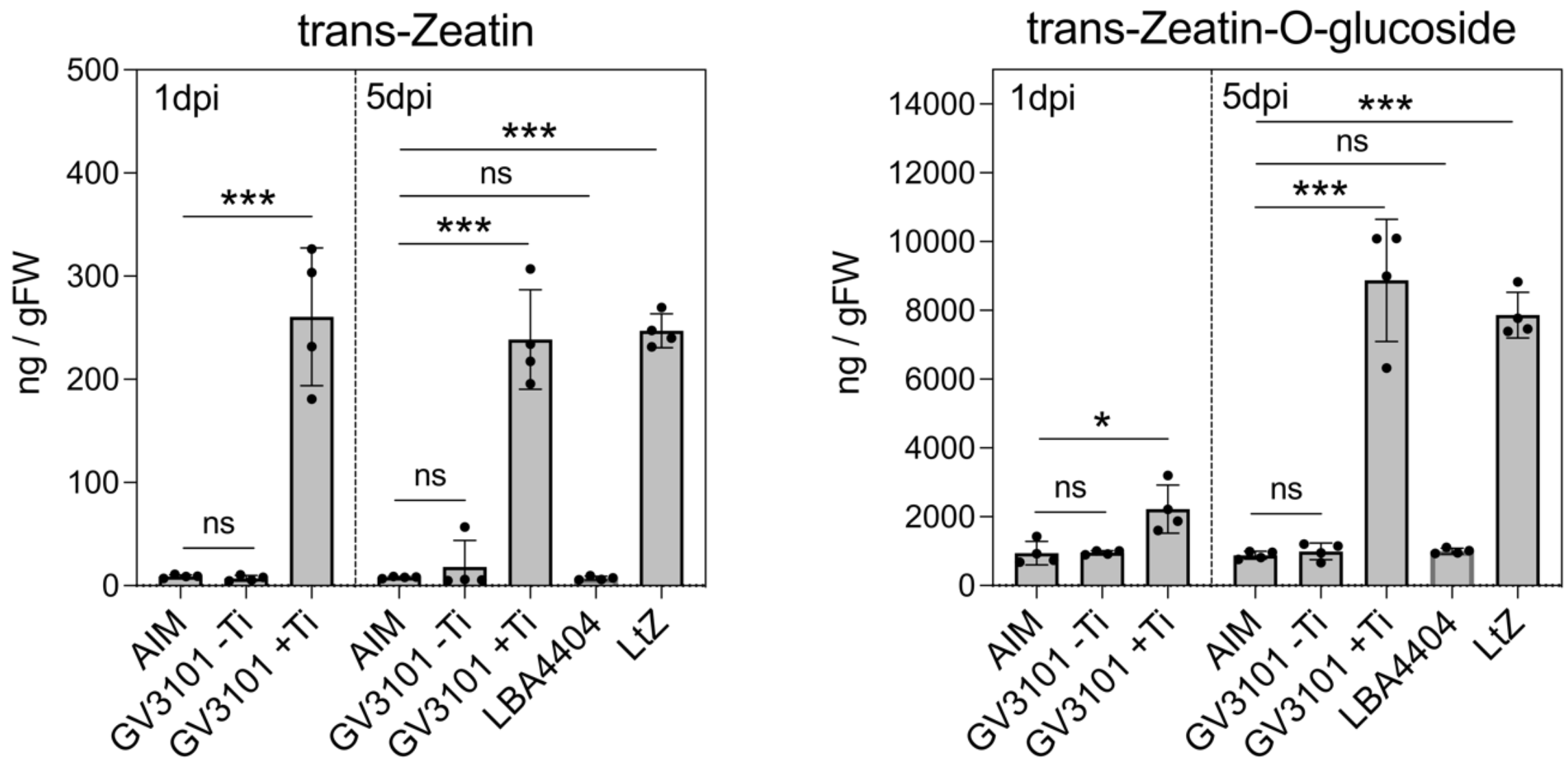

Supplementary Figure 4. trans-Zeatin and trans-Zeatin-O-glucoside levels after *Agrobacterium* infiltration (*n* = 4 biological replicates). Pairwise comparisons were conducted using Student's *t*-test (*p < 0.05, ***p < 0.001; ns = not significant). Error bars represent standard deviation. AIM, *Agrobacterium* infiltration medium; Ti, refers to pMP90; LtZ, refers to the LBA4404 strain transformed with a vector containing the *trans-zeatin synthase* (*tzs*) gene.

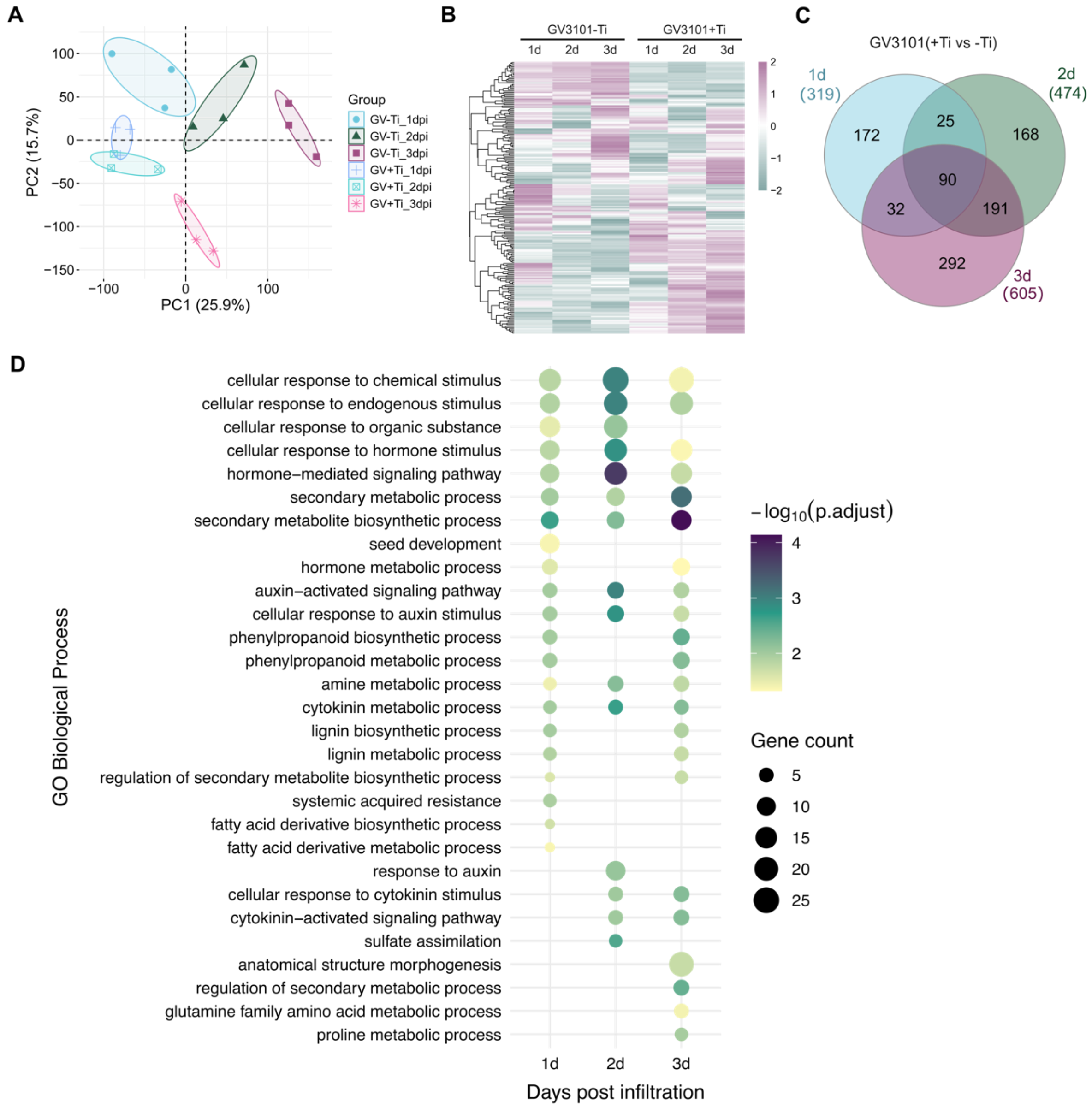

Supplementary Figure 5. (A) Comparative transcriptome analysis of infiltrated *N. benthamiana* leaves with *Agrobacterium* GV3101 -Ti and GV3101 +Ti. **(A)** Principal component analysis (PCA) plot of RNA sequenced samples 1 day (d), 2 d, and 3 d after infiltration using the two different strains. **(B)** Heatmap illustrating the top 200 most variable genes across the samples. Average FPKM values of the three biological replicates were converted to z-score values for plotting. **(C)** Venn diagram depicting the differential expressed genes (DEGs) between GV3101-Ti versus GV3101+Ti, with a $|\log_2$ fold change$|$ [$\log_{2FC}$] > 1.5 and *p* adjusted < 0.05. Detailed information about the DEGs can be found in Supplementary Tables 1-5. **(D)** GO term enrichment dynamics across the first three days

post infiltration. Analysis conducted over Biological Process ontology and a *p* adjusted < 0.05.

Materials and Methods

Agrobacterium strains

Agrobacterium strains GV3101(pMP90), LBA4404, EHA105, AGL-0, AGL-1 were obtained from commercial providers. The pMP90 Ti plasmid was eliminated from GV3101 following the protocol of Hynes et al. (1985). Briefly, the GV3101 bacteria harboring the Ti plasmid pMP90 were grown on LB agar medium containing 50 mg ml^{-1} Rifampicin for three days at 40°C, followed by two days at 28°C. Single colonies were streaked onto both Rifampicin-Gentamicin and Rifampicin-only plates. The LBA4404 strain, transformed with a binary vector containing the *trans-zeatin synthase* gene (*tzs*) was kindly provided by Dr. Jessica Erickson (IPB, Halle) (Erickson et al., 2014).

Infiltration of N. benthamiana leaves

Different *Agrobacterium* strains were grown on LB agar medium containing the respective antibiotics for two days and resuspended in *Agrobacterium* infiltration medium (AIM: 10 mM MES, 10 mM $MgCl_2$, 10 µM acetosyringone). Cultures were adjusted to OD_{600} of 0.2 and incubated for two hours at room temperature. Cultures were then infiltrated into leaves of five-week-old *Nicotiana benthamiana* plants. For cytokinin infiltration, each phytohormone (*trans*-Zeatin, kinetin, and 6-benzylaminopurine (BAP), from Duchefa Biochemie B.V. Haarlem, The Netherlands) was first dissolved in 0.5 mL of 1 N NaOH and subsequently in water for 100 µg ml^{-1} working solutions. Plants were infiltrated three times, every other day. Multiple CK infiltrations were required to produce clear effects, suggesting that continuous CK supply—as provided by ongoing bacterial *tzs* expression—is key for full trichome morphogenesis.

The vector pAGM50583 for betalain biosynthesis (Grützner et al., 2021) was used to help visualize the trichomes, which appear white on a red background.

Trichome counting and imaging

Leaf discs were excised from infiltrated spots using a cork borer (diameter 1 cm). Trichomes were manually counted on 1-cm diameter leaf discs with the VHX-6000 microscope in combination with a VH-Z20 T zoom lens (Keyence, Osaka, Japan). For trichome counting on adaxial and abaxial sides at different leaf developmental stages, leaf discs of infiltrated areas were dipped in a 0.1% w/v Rhodamine B (Sigma) solution for 5 min. Images of the fluorescent trichomes were acquired using a Zeiss Axio Zoom.V16 microscope (excitation 559-585; emission 600-690 nm). Images shown in Figure 1B, 1F were obtained by cutting 1.5 mm wide leaf strips and rotating them 90°. Environmental scanning electron microscopy images were recorded using an ESEM XL-30 FEG (FEI/Philips, Eindhoven, The Netherlands) at specified time points after agroinfiltration.

Scanning electron microscopy

Leaf samples were excised into approximately 5 × 5 mm pieces near the infiltration site and immediately fixed in 3% glutaraldehyde prepared in 0.1 M sodium cacodylate buffer (pH 7.2) for 2 h. Following fixation, samples were rinsed in 0.1 M sodium cacodylate buffer for 10 min and dehydrated through a graded ethanol series (30%, 50%, 70%, 90%, 95%, and 100%). For critical point drying (CPD), samples were incubated in an ethanol/acetone mixture (1:1, v/v) for 45 min, followed by 100% acetone for at least 2 h prior to drying using a critical point dryer. Dried samples were mounted onto SEM stubs using double-sided adhesive tape.
Scanning electron microscopy (SEM) imaging was performed using a Zeiss Gemini 500 at an accelerating voltage of 1 kV using secondary electron (SE) detection. Images were acquired at stage tilt angles of 0° and 40–45°.

Acylsugar profiling

Leaf surface extracts were obtained by dipping one 1-cm-diameter leaf disc into 0.5 mL of 80% methanol solution and inverting the tubes for 2 min. Representative acylsugars (AS) produced by *Nicotiana benthamiana* glandular trichomes were quantified by LC-MS/MS. Metabolites were separated with an Acquity UPLC (Waters Inc.) with a Nucleoshell RP18 column (Macherey & Nagel, 150 mm × 2 mm × 2.7 µm). Solvents and gradient were adjusted according to Balcke et al. (2024). Mass spectrometric analysis was performed by MS1 full scan from 65-1500 Daltons and 100 ms accumulation time (ZenoToF 7600, Sciex GmbH, Darmstadt, Germany) operating in negative mode and controlled by Sciex OS software (Sciex). The identity of specific AS was confirmed by MS/MS spectral matching to an inhouse library. MS1 AS peak areas were determined with Multiquant 3 software (Sciex GmbH, Darmstadt, Germany).

Cytokinin measurements

Cytokinin quantification was performed using 50 mg of homogenized plant tissue extracted with 400 µl of 80% (v/v) methanol containing 25 pg.µl^{-1} *trans*-zeatin-[d5] and 50 pg.µl^{-1} *trans*-zeatin-O-glucoside-[d5] as stable isotope-labeled internal standards. Extracts were centrifuged twice, and the supernatants were collected for analysis. A 10 µl aliquot was injected into an ACQUITY UPLC system (Waters Inc.) equipped with a Nucleodur Gravity C18 SB column (1.8µm EC 100/2 Macherey Nagel, Dueren, Germany). The mobile phase consisted of solvent A (0.1% (v/v) aqueous formic acid) and solvent B (acetonitrile / 0.1% (v/v) formic acid). The gradient was as follows: 98% A for 2 minutes; a linear gradient from 98% to 5% A from 2 to 14 minutes; isocratic 5% A from 14 to 17.5 minutes; isocratic 98% A from 18 to 20 minutes. The flow rate was 0.5 ml min^{-1}, and the column temperature was maintained at 40°C. Analytes were detected using a Sciex TripleQuad 7500+ mass spectrometer (AB Sciex, Darmstadt, Germany.operating in positive electrospray

ionization mode with scheduled multiple reaction monitoring (MRM). Peaks were automatically integrated by the MQ4 algorithm embedded in the Sciex OS 4.0 software (AB Sciex, Darmstadt, Germany), and integration was manually adjusted if necessary. Quantification of trans-zeatin and trans-zeatin-O-glucoside was performed based on the ratio of analyte to corresponding internal standard peak areas.

RNA extraction and sequencing

Total RNA was extracted from *N. benthamiana* leaf discs infiltrated with the different *Agrobacterium* strains, using the RNeasy© Plant Mini Kit (Qiagen United States, 74904) and following the manufacturer's instructions. RNA integrity (RIN > 7.0) was evaluated with BioAnalyzer utilizing the RNA 6000 Nano kit (Agilent Technologies). Library preparation and mRNA sequencing was conducted by the company Novogen UK (www.novogene.com), using Illumina NovaSeq 6000 Sequencing PE150, Q30 ≥85%. Reads were mapped to the *N. benthamiana* genome (Nbe_v1.1) (Kurotani et al., 2023) using the nf-core/rnaseq pipeline (Ewels et al., 2020) with the default parameters. Differential expression analysis was performed using DESeq2 (Love et al., 2014). PCA and heatmap were plotted using the SRplot website (Tang et al., 2023). Venn diagram was constructed using InteractiVenn (Heberle et al., 2015). GO term enrichment analysis was conducted in RStudio 2026.01.0 using ClusterProfiler package. Raw sequencing reads have been deposited in the NCBI Bioproject accession PRJNA1424681.

Statistical analysis

Statistical analyses were performed using GraphPad Prism 9.5.1 software (https://www.graphpad.com/). Pairwise comparisons were conducted using Student's *t*-test with a significance threshold of $p < 0.05$. For multiple group comparisons, one-way ANOVA was used followed by Tukey's post hoc test, with $p < 0.05$ considered significant.

References

Balcke, G.U., Vahabi, K., Giese, J., Finkemeier, I., and Tissier, A. (2024). Coordinated metabolic adaptation of Arabidopsis thaliana to high light. The Plant Journal **120**:387-405. https://doi.org/10.1111/tpj.16992.

Erickson, J.L., Ziegler, J., Guevara, D., Abel, S., Klösgen, R.B., Mathur, J., Rothstein, S.J., and Schattat, M.H. (2014). Agrobacterium-derived cytokinin influences plastid morphology and starch accumulation in Nicotiana benthamiana during transient assays. BMC Plant Biology **14**:127. 10.1186/1471-2229-14-127.

Ewels, P.A., Peltzer, A., Fillinger, S., Patel, H., Alneberg, J., Wilm, A., Garcia, M.U., Di Tommaso, P., and Nahnsen, S. (2020). The nf-core framework for community-curated bioinformatics pipelines. Nature Biotechnology **38**:276-278. 10.1038/s41587-020-0439-x.

Grützner, R., Schubert, R., Horn, C., Yang, C., Vogt, T., and Marillonnet, S. (2021). Engineering Betalain Biosynthesis in Tomato for High Level Betanin Production in Fruits. Frontiers in Plant Science **12**:682443.

Heberle, H., Meirelles, G.V., da Silva, F.R., Telles, G.P., and Minghim, R. (2015). InteractiVenn: a web-based tool for the analysis of sets through Venn diagrams. BMC Bioinformatics **16**:169. 10.1186/s12859-015-0611-3.

Hynes, M.F., Simon, R., and Pühler, A. (1985). The development of plasmid-free strains of Agrobacterium tumefaciens by using incompatibility with a Rhizobium meliloti plasmid to eliminate pAtc58. Plasmid **13**:99-105. https://doi.org/10.1016/0147-619X(85)90062-9.

Kurotani, K.-i., Hirakawa, H., Shirasawa, K., Tanizawa, Y., Nakamura, Y., Isobe, S., and Notaguchi, M. (2023). Genome Sequence and Analysis of Nicotiana benthamiana, the Model Plant for Interactions between Organisms. Plant and Cell Physiology **64**:248-257. 10.1093/pcp/pcac168.

Love, M.I., Huber, W., and Anders, S. (2014). Moderated estimation of fold change and dispersion for RNA-seq data with DESeq2. Genome Biology **15**:550. 10.1186/s13059-014-0550-8.

Tang, D., Chen, M., Huang, X., Zhang, G., Zeng, L., Zhang, G., Wu, S., and Wang, Y. (2023). SRplot: A free online platform for data visualization and graphing. PLoS One **18**:e0294236. 10.1371/journal.pone.0294236.
