# Supplementary material for: Cytokinin-mediated trichome initiation in Nicotiana benthamiana following Agrobacterium tumefaciens infiltration
Source: Plant Commun. 2026 Jun 17;7(10):101971. doi: 10.1016/j.xplc.2026.101971 (PMC13681608; doi:10.1016/j.xplc.2026.101971)
Supplement: Supplementary file 2 [file mmc3.pdf]

**Plant Communications**
Correspondence

CellPress
Partner Journal

# Cytokinin-mediated trichome initiation in *Nicotiana benthamiana* following *Agrobacterium tumefaciens* infiltration

Dear Editor,

*Agrobacterium tumefaciens* is widely used in plant genetic engineering owing to its tumor-inducing (Ti) plasmid, which contains a transferable T-DNA region and a virulence (*vir*) region encoding a type IV secretion system (Lacroix and Citovsky, 2019). In biotechnology, disarmed strains lacking oncogenes are routinely used together with binary vectors that carry genes of interest for stable or transient transformation. *Agrobacterium*-mediated leaf infiltration of *Nicotiana benthamiana* is an efficient method for transient gene expression. One commonly used disarmed strain is GV3101, which harbors the Ti plasmid pMP90. Within the *vir* region, this plasmid contains the *trans-zeatin synthase* (*tzs)* gene, which triggers the production and release of the cytokinin (CK) *trans*-zeatin (*t*-zeatin) upon infection (Beaty et al., 1986). Exogenous CK application mimics several effects observed with GV3101(pMP90) (hereafter referred to as GV3101+Ti), including induction of stromules, repositioning of plastids around the nucleus, and accumulation of soluble sugars and starch (Erickson et al., 2014).

While performing routine transient expression experiments with GV3101+Ti, we observed a striking macroscopic phenotype: a marked increase in leaf trichome density within the infiltrated areas. This effect became visible to the naked eye approximately 7 days post-infiltration (dpi) and was absent in leaves infiltrated with EHA105 or AGL-1 (Figure 1A). The induced trichomes were glandular trichomes (GTs) that produce and secrete acylsugars (ASs), sticky specialized metabolites involved in stress protection. Here, we demonstrate that GV3101+Ti triggers *de novo* GT formation in *N. benthamiana* leaves and that this response is mediated by CK production from the pMP90 Ti plasmid.

To determine whether trichome induction is specific to GV3101 + Ti, we infiltrated *N. benthamiana* leaves with GV3101+Ti and several other commonly used strains, including LBA4404, EHA105, AGL-0, and AGL-1. At 10 dpi, GV3101+Ti induced approximately 3-fold higher trichome density in the infiltrated areas than in untreated or mock-infiltrated controls, whereas the other strains did not increase trichome density (Figures 1B and 1C; Supplemental Figure 1A). By 14 dpi, some strains, particularly AGL-0, caused yellowing and senescence in the infiltrated tissue, whereas GV3101+Ti areas remained green (Supplemental Figure 1A). Microscopic time-course observations revealed progressive trichome initiation following GV3101+Ti inoculation, starting as early as 2 dpi, when pavement cells began to bulge and round glandular tips emerged. By 5 dpi, clearly elongated trichomes were visible (Figure 1D; Supplemental Figure 3). Scanning electron microscopy (SEM) further showed that new trichomes frequently emerged from neighboring cells or even from the basal cells of existing trichomes (Figure 1D), indicating that *de novo* initiation can occur in different epidermal cell types. The extent of trichome induction varied among leaves and was more pronounced in younger leaves and on the adaxial surface (Supplemental Figure 2). Trichome development was further assessed by quantifying AS levels, revealing an approximately 2-fold increase in GV3101+Ti-infiltrated samples (Figure 1E). To test whether trichome induction was caused by the presence of the pMP90 plasmid or the GV3101 chromosomal background, we generated a Ti plasmid-cured GV3101 strain (GV3101-Ti). Trichome density and AS levels in GV3101-Ti-infiltrated areas remained comparable to those in the controls (Figures 1B, C, and 1E). A key distinguishing feature of pMP90 is the presence of the *tzs* gene. Consistent with this, *t*-zeatin levels rose within 24 h after infiltration with GV3101+Ti and remained elevated at 5 dpi, accompanied by an accumulation of *t*-zeatin-O-glucoside, an inactive form of *t*-zeatin (Supplemental Figure 4). An LBA4404 strain transformed with a binary vector carrying *tzs* (LtZ) (Erickson et al., 2014) induced trichome densities comparable to those induced by GV3101+Ti and doubled AS levels, demonstrating that CK production via *tzs* is sufficient for this effect (Figures 1B, 1C, and 1E; Supplemental Figure 4). Similarly, a C58 strain harboring the *tzs* gene within its Ti plasmid was recently reported to induce trichome formation, yielding trichome numbers comparable to those induced by GV3101+Ti (Chen et al., 2026). Direct infiltration of synthetic CKs (*t*-zeatin, kinetin, and 6-benzylaminopurine [BAP]) reproduced the phenotype, although to a lesser extent: *t*-zeatin and BAP each caused an approximately 2-fold increase in trichome density and elevated AS levels, whereas kinetin produced a smaller but significant trichome increase without significantly affecting AS content (Figures 1F–1H).

CKs regulate cell division and leaf expansion and cooperate with auxin in controlling meristem formation and maintenance. Although the roles of jasmonic acid and auxin in trichome development are well established, the contribution of CK signaling remains less explored. In *Arabidopsis*, which bears only non-GTs, ectopic expression of the *Agrobacterium isopentenyl transferase* gene, which encodes a CK biosynthesis enzyme, induced trichome formation on carpels (Greenboim-Wainberg et al., 2005). In tomato, which produces both GTs and non-GTs, exogenous BAP specifically induced GTs, including types with uni- and multicellular heads, whereas jasmonic acid had broader effects across all trichome types (Maes and Goossens, 2010). These studies highlight the distinct regulatory mechanisms by which CKs and other phytohormones control different trichome types and their associated metabolism across species.

 © 2026 The Author(s). Published by Elsevier Inc. on behalf of CAS Center for Excellence in Molecular Plant Sciences, Chinese Academy of Sciences, and Chinese Society for Plant Biology. This is an open access article under the CC BY license (http://creativecommons.org/licenses/by/4.0/). 

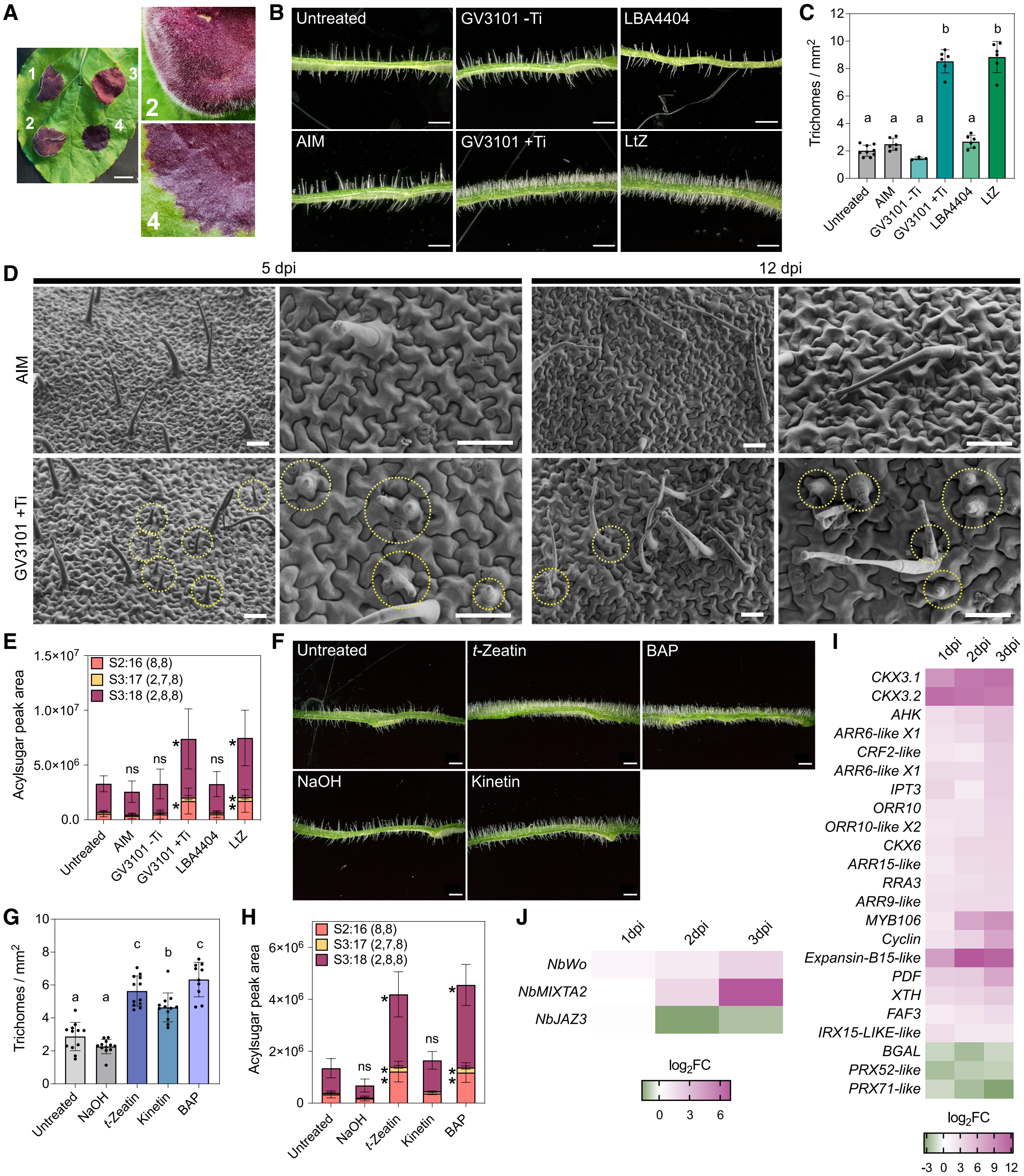


**Figure 1. Cytokinin-mediated trichome development in *Nicotiana benthamiana* following *Agrobacterium tumefaciens* infiltration.**

**(A)** A leaf of *N. benthamiana* infiltrated with three different *Agrobacterium* strains harboring a vector for betalain biosynthesis, 4 days post-infiltration (dpi). Spots 1 and 2: GV3101(pMP90); 3: EHA105; and 4: AGL-1. The right image shows a close-up of infiltrated spots 2 and 4 at 7 dpi. Scale bar: 1 cm.

**(B)** Cross-sections of infiltrated leaf spots at 14 dpi showing trichomes on the adaxial and abaxial sides (See Supplemental Figure 1A for other *Agrobacterium* strains). AIM, *Agrobacterium* infiltration medium. Ti refers to pMP90, and LtZ refers to strain LBA4404 transformed with a vector containing the *trans-zeatin synthase* (*tzs*) gene. Scale bar: 1 mm.

**(C)** Trichome density on the adaxial side of infiltrated spots corresponding to the treatments depicted in **(B)** at 10 dpi ($n$ = 3–9 biological replicates).

**(D)** Scanning electron microscopy images of trichome initiation at 5 and 12 dpi days after infiltration with mock solution (AIM) or GV3101+Ti. Yellow circles indicate newly emerging trichomes. Scale bar: 100 μm.

**(E)** Quantification of the main acylsugars (ASs) using liquid chromatography–tandem mass spectrometry (LC–MS/MS) ($n$ = 6 biological replicates). The nomenclature of ASs is as follows: S, sucrose (sugar core), followed by the number of acyl chains. The number after the colon indicates the total number of carbons in all acyl chains, and the numbers in parentheses indicate the number of carbons in each acyl chain, separated by commas.

**(F)** Infiltration of different CKs mimicked GV3101+Ti trichome formation in *N. benthamiana*. Images were taken at 14 dpi. *t*-zeatin, *trans*-zeatin; BAP, 6-benzylaminopurine.

**(G)** Trichome density on the adaxial side of infiltrated spots corresponding to the treatments depicted in **(F)** ($n$ = 10–12 biological replicates).

*(legend continued on next page)*

To gain deeper insight into the molecular basis of *Agrobacterium*- and CK-induced trichome formation, we performed RNA sequencing (RNA-seq) on leaves infiltrated with GV3101-Ti *vs.* GV3101+Ti at 1, 2, and 3 dpi. Principal-component and differential expression analyses revealed a gradual divergence between the two strain treatments over time (Supplemental Figures 5A and S5B). The number of differentially expressed genes (DEGs) increased over time. Using thresholds of $|\log_2 \text{fold change}| > 1.5$ and adjusted $p < 0.05$, we identified 319, 474, and 605 DEGs at 1, 2, and 3 dpi, respectively (Supplemental Figures 5B and S5C; Supplemental Tables 1–5). Gene Ontology enrichment analysis of DEGs revealed significant overrepresentation of terms related to cellular responses to hormone stimulus and hormone-mediated signaling pathways (Supplemental Figure 5D). Among the top DEGs were multiple CK catabolic genes (e.g., CK dehydrogenase/oxidases (CKXs)) as well as core components of the CK signaling pathway, including *histidine kinase* (*AHK*)-like genes and several *Arabidopsis response regulator*-like genes (*ARRs*) (Figure 1I; Table S6).

Trichomes are epidermal outgrowths. In *N. tabacum* and other species, multicellular GTs arise from enlargement of protodermal cells, followed by asymmetric cell divisions (Tissier, 2012). Thus, trichome morphogenesis involves dynamic cell wall remodeling, beginning with wall loosening and followed by wall thickening. Consistent with this, a gene annotated as *expansin B15-like* was significantly upregulated as early as 24 h after infiltration with GV3101+Ti (Figure 1I; Table S6). Expansins are positively regulated by auxin and CK and play a key role in cell wall loosening, thereby promoting cell expansion (Samalova et al., 2024). Furthermore, genes encoding several peroxidases (*PRX*), an xyloglucan endotransglucosylase/hydrolase, and a protodermal factor 1-like protein were differentially expressed across the three time points, supporting roles for cell wall remodeling and expansion in trichome morphogenesis. To our knowledge, the genes that link CK signaling and trichome development have not been characterized in tobacco. We observed consistent upregulation from 1 dpi onward of a *cyclin* gene, a *growth regulating factor* gene, and the *MIXTA-like* transcriptional regulator gene *MYB106*, which was previously implicated in GT initiation and trichome branching in tomato (Li et al., 2026) (Figure 1I; Supplemental Tables 5 and 6). The HD-zip transcription factor Woolly (Wo), a master regulator of multicellular trichome morphogenesis in both tomato and tobacco, displayed progressive upregulation from 1 dpi (Figure 1J; Supplemental Tables 7 and 8) (Wu et al., 2020, 2023). This upregulation coincided with marked downregulation of *NbJAZ3*, which encodes a repressor of *NbWo*, and strong induction of its target, the *NbMIXTA2* transcription factor (Yan et al., 2022), which showed an approximately 7-fold increase at 3 dpi (Figure 1J). *NbMIXTA2* shares high sequence similarity with the tomato *MX1* (Supplemental Table 8), a key determinant of cell fate in capitate trichomes, which are analogous to the GTs of *N. benthamiana* (Wu et al., 2023). Notably, both *NbWo* and *NbMIXTA2* are induced later (primarily at 3 dpi) than the early CK-responsive genes, indicating that trichome development genes are likely indirect targets of CK signaling. Numerous additional candidate regulators showed significant expression changes at one or more time points; their potential roles in trichome morphogenesis warrant further investigation (Supplemental Table 7).

In conclusion, CK-producing *Agrobacterium* strains induce GT initiation and AS accumulation in *N. benthamiana*. Transcriptional changes induced by GV3101+Ti infiltration support a role for CKs in triggering *de novo* trichome morphogenesis from epidermal cells. However, whether cell cycle re-entry is required for this process remains unresolved. This trichome induction phenomenon, which may have gone unnoticed despite the widespread use of GV3101(pMP90) in plant laboratories, provides a novel experimental system for dissecting the molecular control of cell fate and the contribution of CKs to trichome development and specialized metabolism. Because *N. benthamiana* leaf infiltration by *Agrobacterium* is routinely employed for a wide range of assays—including pathway reconstitution and disease resistance studies—we recommend routinely comparing infiltration with GV3101(pMP90) and a non-CK-producing strain such as LBA4404 to determine whether CK production, GT induction, or AS accumulation influences experimental outcomes.

## FUNDING

This work was funded in part by the *KETCHUP* project of the Leibniz Association (project number K287/2019) and by core funds of the Leibniz Institute of Plant Biochemistry.

## ACKNOWLEDGMENTS

The authors thank Dr. Jessica Erickson for kindly providing the *Agrobacterium* LtZ strain. The authors also thank Dr. Jörg Ziegler for his assistance with cytokinin measurements. No conflict of interest is declared.

## AUTHOR CONTRIBUTIONS

R.S. and A.B. performed most of the experiments and analyzed the data; A.B. analyzed the RNA-seq data and wrote the manuscript draft; D.E. mapped RNA-seq reads; G.U.B. measured AS; F.S. performed ESEM imaging; S.K. prepared the samples for SEM and acquired SEM images; C.H. generated the cured GV3101 *Agrobacterium* strain and performed infiltrations with vectors for betalain production; S.M. first observed the trichome phenotype and designed the experiments; and A.T. acquired funding, supervised the project, and revised the manuscript. All authors read and approved the manuscript.

## DECLARATION OF GENERATIVE AI AND AI-ASSISTED TECHNOLOGIES IN THE WRITING PROCESS

During the preparation of this work, the authors used Grok (grok.com) solely to improve the grammar and style of the manuscript. No scientific content was drawn from the use of AI. The authors reviewed and edited the output as needed and take full responsibility for the content of the published article.

**(H)** Quantification of the main ASs using LC–MS/MS corresponding to the treatments depicted in **(F)** ($n = 3$ biological replicates).

**(I)** Heatmap of selected differentially expressed genes (DEGs) between GV3101+Ti and GV3101-Ti, with a $|\log_2 \text{fold change} (\log_2\text{FC})| > 1.5$ and adjusted $p < 0.05$. For gene IDs and actual expression values, see Supplemental Table 6.

**(J)** $\log_2$FC (GV3101+Ti versus GV3010-Ti) of known molecular regulators of trichome initiation in tobacco. For gene IDs and actual expression values, see Supplemental Table 8. Different letters indicate significant differences based on one-way ANOVA ($p < 0.05$, with Tukey's correction). Pairwise comparisons of individual ASs were conducted relative to the untreated samples, and significance was assessed using Student's *t*-test (*$p < 0.05$ and ns, non-significant). Error bars show standard deviation.

## SUPPLEMENTAL INFORMATION

Supplemental information is available at *Plant Communications Online*.

Received: May 5, 2026
Revised: June 4, 2026
Accepted: June 13, 2026
Published: June 17, 2026

*Alejandro Brand[1,5], Robert Säbel[1,5], Dario Esposto[1], Gerd U. Balcke[1], Frank Syrowatka[2], Stephanie Krüger[3], Claudia Horn[1], Sylvestre Marillonnet[1] and Alain Tissier[1,4,*]*

[1]Leibniz Institute of Plant Biochemistry, Department of Cell and Metabolic Biology, Weinberg 3, 06120 Halle (Saale), Germany
[2]Martin-Luther-University Halle-Wittenberg, Interdisziplinäres Zentrum für Materialwissenschaften, Nanotechnikum Weinberg, Heinrich-Damerow-Str. 4, 06120 Halle (Saale), Germany
[3]Martin-Luther-University Halle-Wittenberg, Core Facility Microscopy, Weinbergweg 22, 06120 Halle (Saale), Germany
[4]Martin-Luther University Halle-Wittenberg, Institute of Pharmacy, 06120 Halle (Saale), Germany
[5]These authors contributed equally to this article.
***Correspondence: Alain Tissier (alain.tissier@ipb-halle.de)**
https://doi.org/10.1016/j.xplc.2026.101971

## REFERENCES

**Beaty, J.S., Powell, G.K., Lica, L., Regier, D.A., MacDonald, E.M.S., Hommes, N.G., and Morris, R.O.** (1986). Tzs, a nopaline Ti plasmid gene from Agrobacterium tumefaciens associated with trans-zeatin biosynthesis. Molec. Gen. Genet. **203**:274–280. https://doi.org/10.1007/BF00333966.

**Chen, J., Hands, P., Patel, M., et al.** (2026). Trichome formation in Nicotiana benthamiana is induced by certain *Agrobacterium tumefaciens* strains. Front. Plant Sci. **17**:1762747. https://doi.org/10.3389/fpls.2026.1762747.

**Erickson, J.L., Ziegler, J., Guevara, D., Abel, S., Klösgen, R.B., Mathur, J., Rothstein, S.J., and Schattat, M.H.** (2014). Agrobacterium-derived cytokinin influences plastid morphology and starch accumulation in Nicotiana benthamiana during transient assays. BMC Plant Biol. **14**:127. https://doi.org/10.1186/1471-2229-14-127.

**Greenboim-Wainberg, Y., Maymon, I., Borochov, R., Alvarez, J., Olszewski, N., Ori, N., Eshed, Y., and Weiss, D.** (2005). Cross Talk between Gibberellin and Cytokinin: The Arabidopsis GA Response Inhibitor SPINDLY Plays a Positive Role in Cytokinin Signaling. Plant Cell **17**:92–102. https://doi.org/10.1105/tpc.104.028472.

**Lacroix, B., and Citovsky, V.** (2019). Pathways of DNA Transfer to Plants from Agrobacterium tumefaciens and Related Bacterial Species. Annu. Rev. Phytopathol. **57**:231–251. https://doi.org/10.1146/annurev-phyto-082718-100101.

**Li, M., Huang, Y., Du, Y., and Wu, S.** (2026). The Genetic Wiring of Plant Trichomes: From Initiation to Fate Specification. Adv. Sci. **13**:e12008. https://doi.org/10.1002/advs.202512008.

**Maes, L., and Goossens, A.** (2010). Hormone-mediated promotion of trichome initiation in plants is conserved but utilizes species and trichome-specific regulatory mechanisms. Plant Signal. Behav. **5**:205–207. https://doi.org/10.4161/psb.5.2.11214.

**Samalova, M., Melnikava, A., Elsayad, K., Peaucelle, A., Gahurova, E., Gumulec, J., Spyroglou, I., Zemlyanskaya, E.V., Ubogoeva, E.V., Balkova, D., et al.** (2023). Hormone-regulated expansins: Expression, localization, and cell wall biomechanics in Arabidopsis root growth. Plant Physiol. **194**:209–228. https://doi.org/10.1093/plphys/kiad228.

**Tissier, A.** (2012). Glandular trichomes: what comes after expressed sequence tags? Plant J. **70**:51–68. https://doi.org/10.1111/j.1365-313X.2012.04913.x.

**Wu, M.-L., Cui, Y.-C., Ge, L., Cui, L.-P., Xu, Z.-C., Zhang, H.-Y., Wang, Z.-J., Zhou, D., Wu, S., Chen, L., and Cui, H.** (2020). NbCycB2 represses Nbwo activity via a negative feedback loop in tobacco trichome development. J. Exp. Bot. **71**:1815–1827. https://doi.org/10.1093/jxb/erz542.

**Wu, M., Chang, J., Han, X., Shen, J., Yang, L., Hu, S., Huang, B.-B., Xu, H., Xu, M., Wu, S., et al.** (2023). A HD-ZIP transcription factor specifies fates of multicellular trichomes via dosage-dependent mechanisms in tomato. Dev. Cell **58**:278–288.e5. https://doi.org/10.1016/j.devcel.2023.01.009.

**Yan, X., Cui, L., Liu, X., Cui, Y., Wang, Z., Zhang, H., Chen, L., and Cui, H.** (2022). NbJAZ3 is required for jasmonate-meditated glandular trichome development in Nicotiana benthamiana. Physiol. Plant. **174**:e13666. https://doi.org/10.1111/ppl.13666.

Plant Communications, Volume 7

## Supplemental information

# Cytokinin-mediated trichome initiation in *Nicotiana benthamiana* following *Agrobacterium tumefaciens* infiltration

**Alejandro Brand, Robert Säbel, Dario Esposto, Gerd U. Balcke, Frank Syrowatka, Stephanie Krüger, Claudia Horn, Sylvestre Marillonnet, and Alain Tissier**

## Supplementary Figures and Materials and Methods

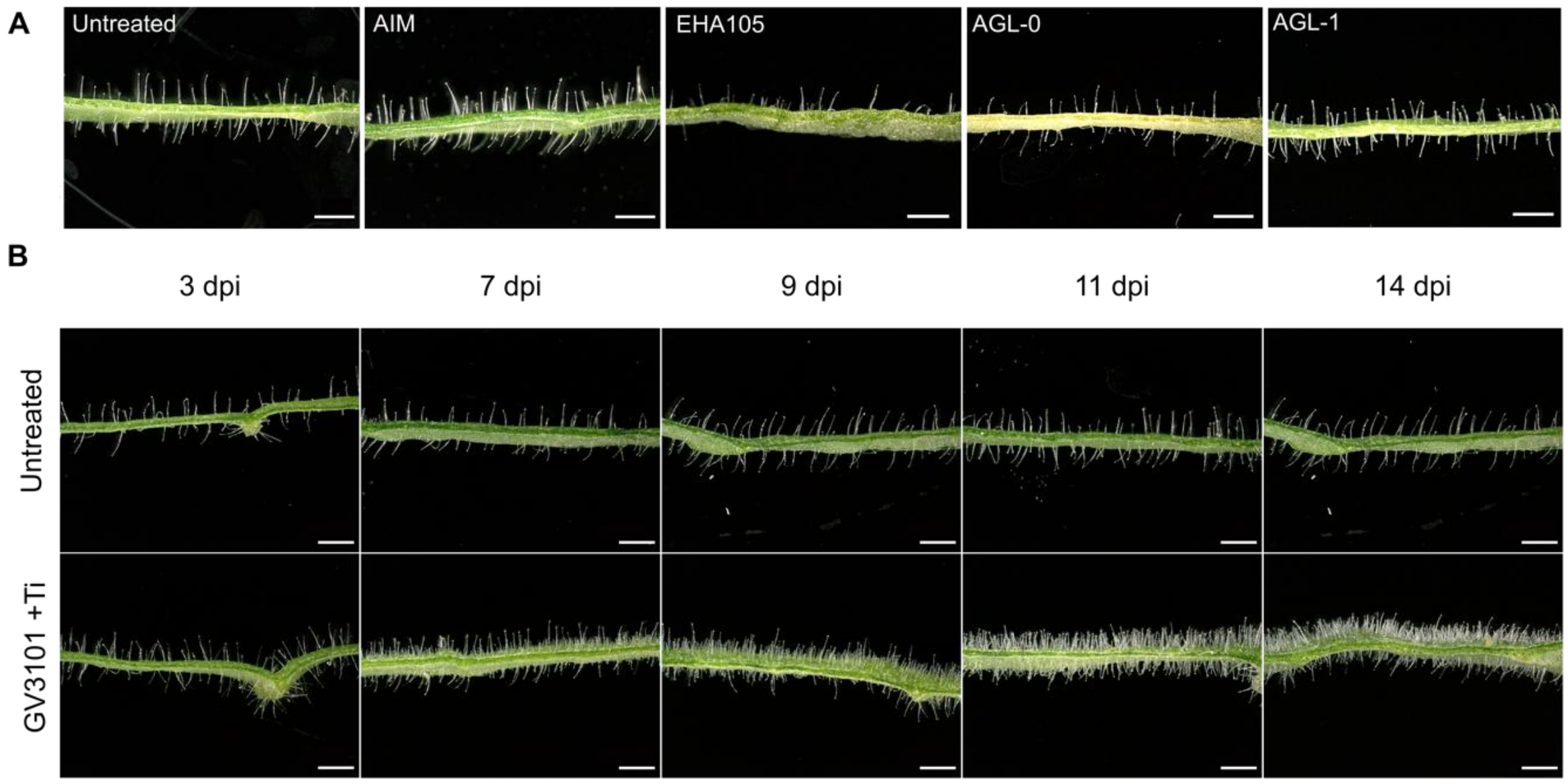


**Supplementary Figure 1. (A)** Trichome development on the adaxial and abaxial sides of leaves of *Nicotiana benthamiana* infiltrated by different *Agrobacterium tumefaciens* strains 14 days post-infiltration (dpi). AIM: Agrobacterium infiltration medium. **(B)** Trichome density over time after infiltration with GV3101 + Ti. Scale bar = 1 mm. Ti, refers to pMP90.

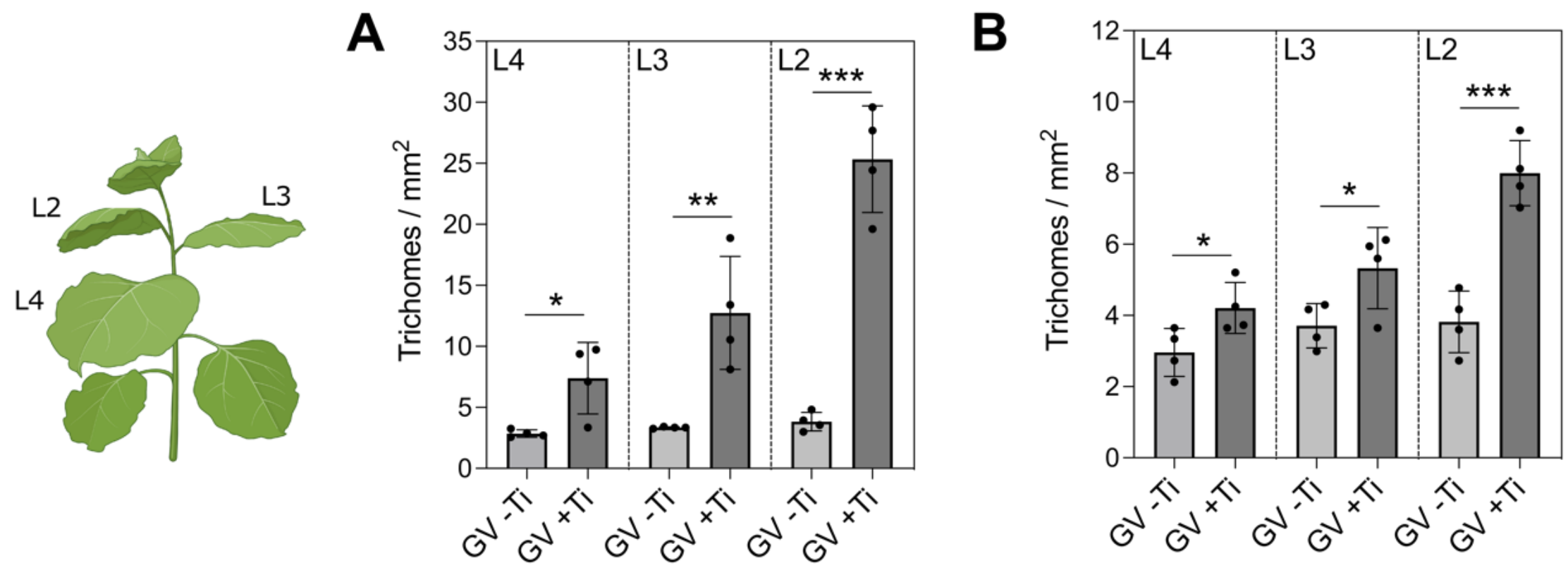


**Supplementary Figure 2.** Trichome counting using leaves of three developmental stages. Trichomes were counted on the adaxial **(A)** and abaxial **(B)** side of the leaves after 12 dpi (*n* = 4 biological replicates). Pairwise comparisons were conducted using Student's *t*-test (*$p < 0.05$, **$p < 0.01$, ***$p < 0.001$). Error bars represent standard deviation. GV: GV3101; Ti: pMP90. Created in BioRender. Brand Duran, A. (2026) https://BioRender.com/pd207hz.

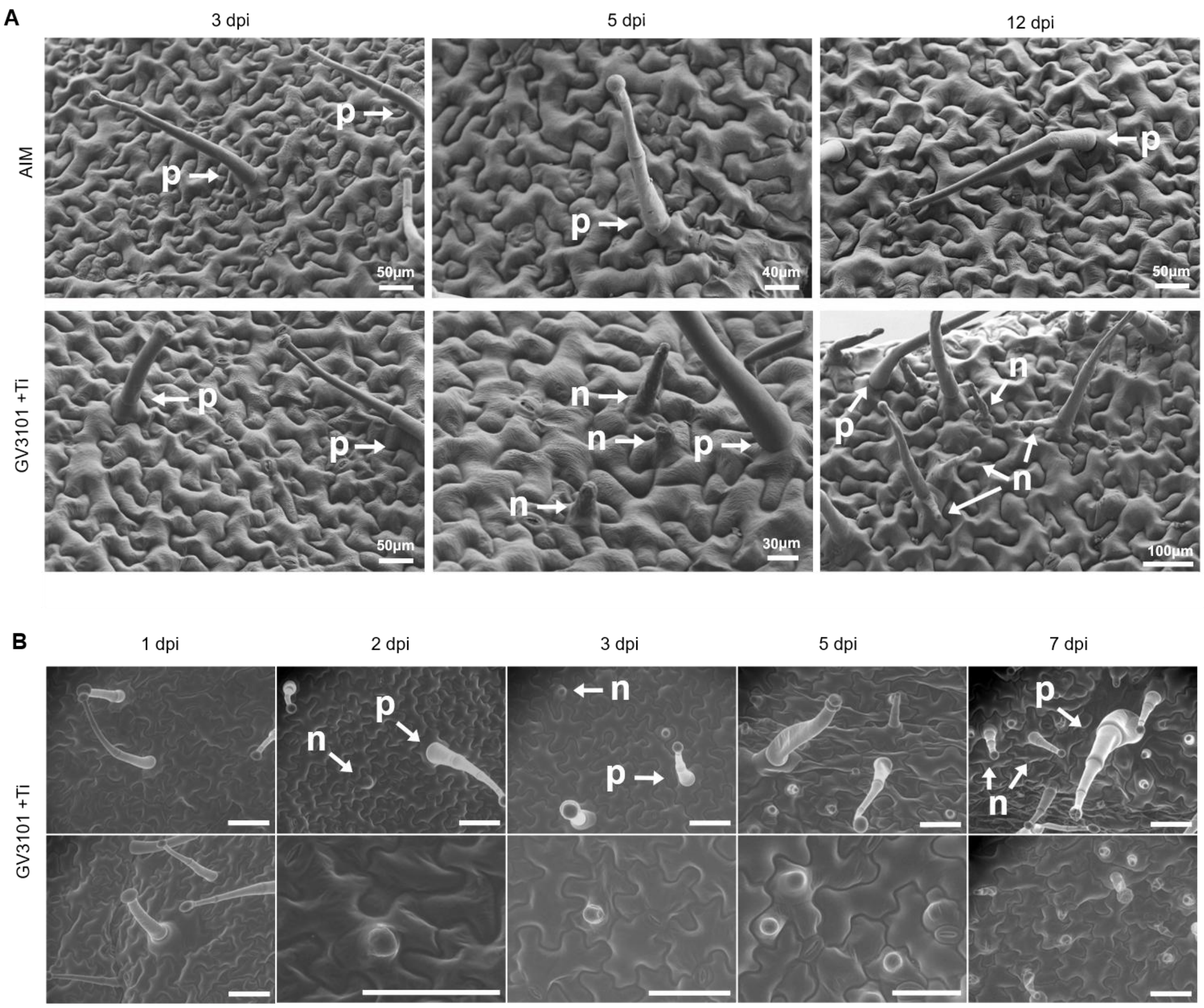


**Supplementary Figure 3. (A)** Scanning electron microscopy (SEM) and **(B)** Environmental SEM of trichome initiation after *Agrobacterium* infiltration. Mock AIM: Agrobacterium infiltration medium; Ti: pMP90 Ti plasmid; p: preexisting trichomes; n: newly emerging trichomes. Scale bar in B: 100 µm.

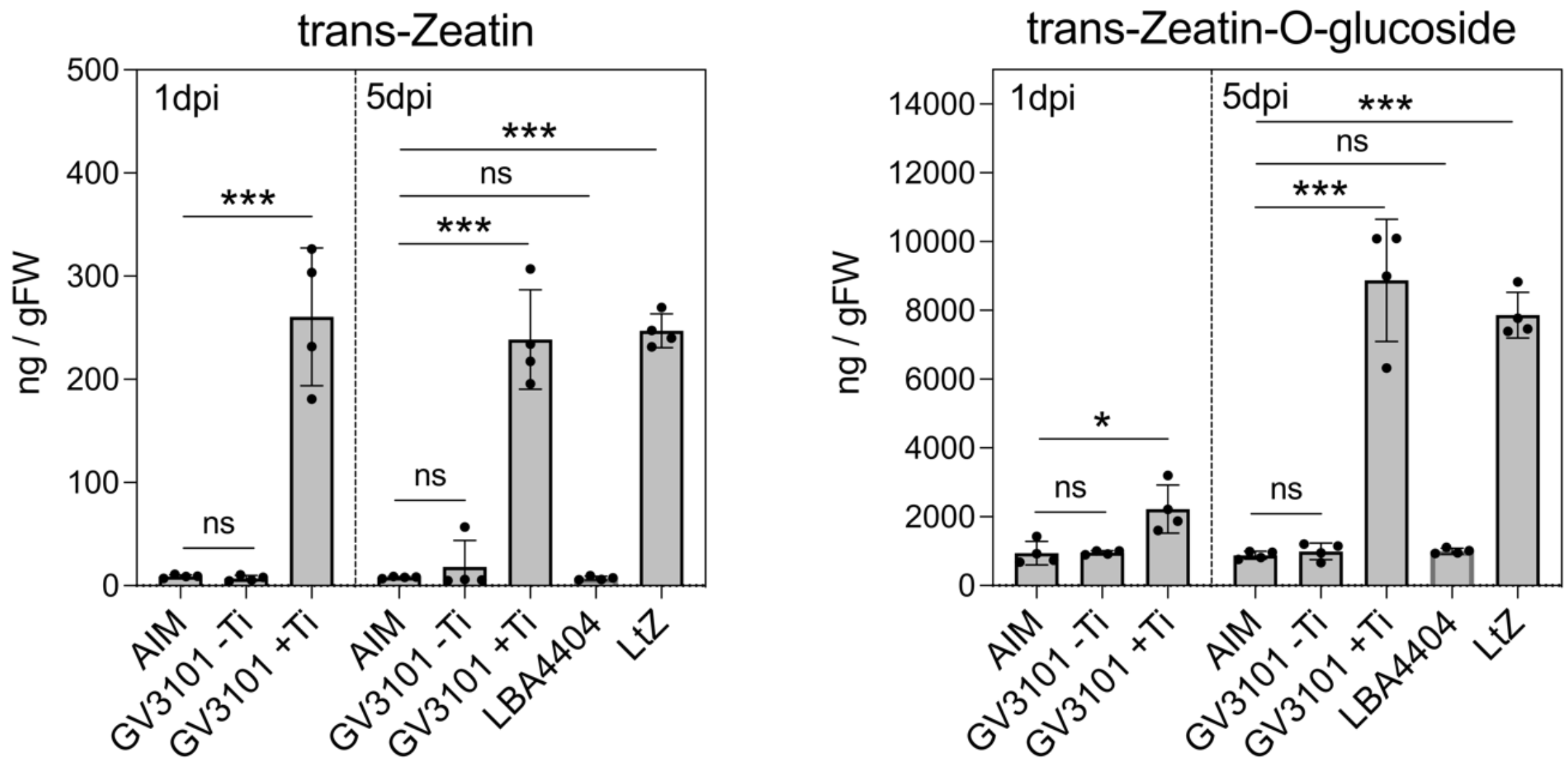


**Supplementary Figure 4.** trans-Zeatin and trans-Zeatin-O-glucoside levels after *Agrobacterium* infiltration (*n* = 4 biological replicates). Pairwise comparisons were conducted using Student's *t*-test (*$p < 0.05$, ***$p < 0.001$; ns = not significant). Error bars represent standard deviation. AIM, *Agrobacterium* infiltration medium; Ti, refers to pMP90; LtZ, refers to the LBA4404 strain transformed with a vector containing the *trans-zeatin synthase* (*tzs*) gene.

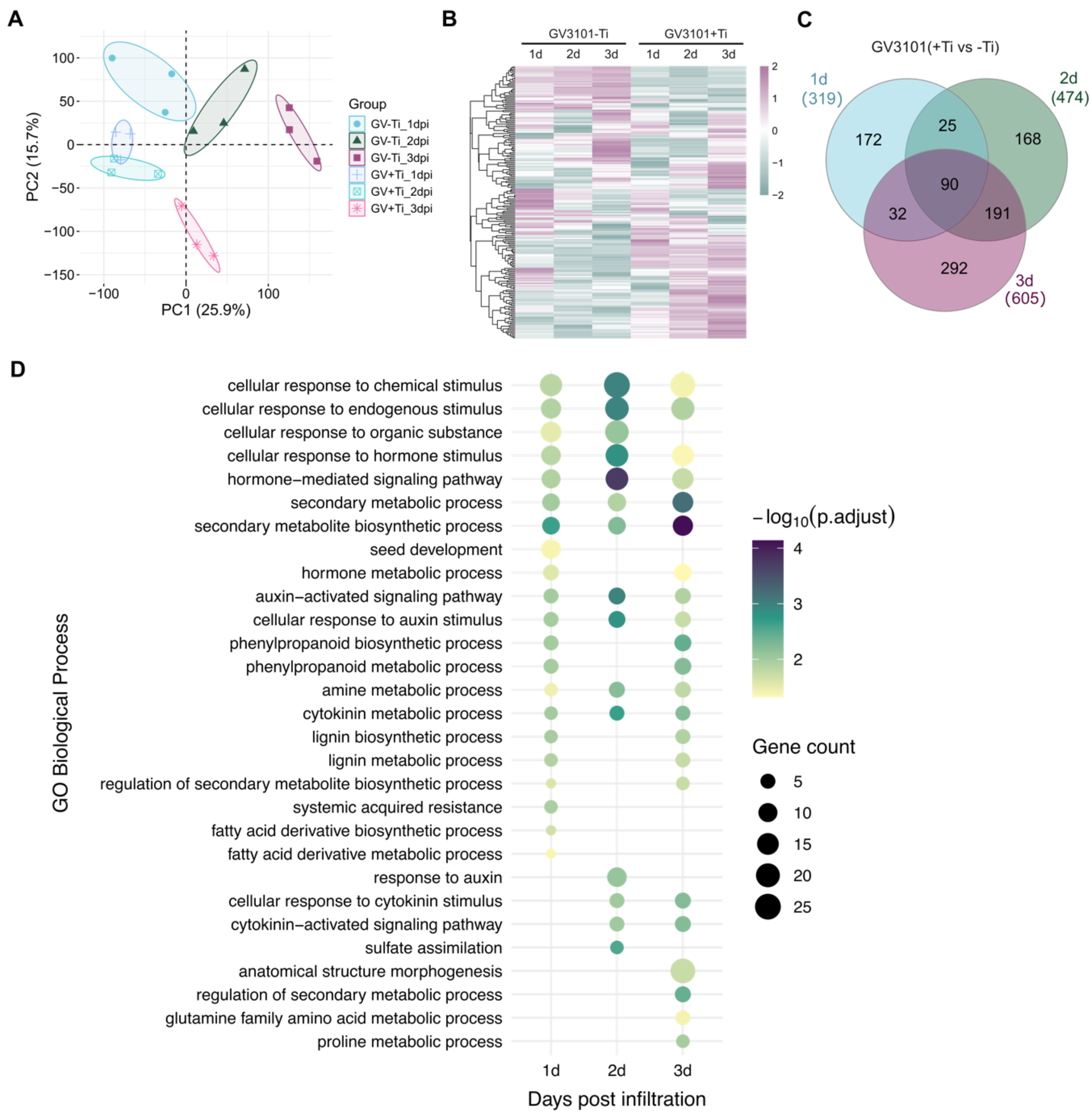


**Supplementary Figure 5. (A)** Comparative transcriptome analysis of infiltrated *N. benthamiana* leaves with *Agrobacterium* GV3101 -Ti and GV3101 +Ti. **(A)** Principal component analysis (PCA) plot of RNA sequenced samples 1 day (d), 2 d, and 3 d after infiltration using the two different strains. **(B)** Heatmap illustrating the top 200 most variable genes across the samples. Average FPKM values of the three biological replicates were converted to z-score values for plotting. **(C)** Venn diagram depicting the differential expressed genes (DEGs) between GV3101-Ti versus GV3101+Ti, with a $|\log_2$ fold change$|$ [$\log_{2FC}$] > 1.5 and *p* adjusted < 0.05. Detailed information about the DEGs can be found in Supplementary Tables 1-5. **(D)** GO term enrichment dynamics across the first three days

post infiltration. Analysis conducted over Biological Process ontology and a *p* adjusted < 0.05.

## Materials and Methods

*Agrobacterium strains*

*Agrobacterium* strains GV3101(pMP90), LBA4404, EHA105, AGL-0, AGL-1 were obtained from commercial providers. The pMP90 Ti plasmid was eliminated from GV3101 following the protocol of Hynes et al. (1985). Briefly, the GV3101 bacteria harboring the Ti plasmid pMP90 were grown on LB agar medium containing 50 mg $ml^{-1}$ Rifampicin for three days at 40°C, followed by two days at 28°C. Single colonies were streaked onto both Rifampicin-Gentamicin and Rifampicin-only plates. The LBA4404 strain, transformed with a binary vector containing the *trans-zeatin synthase* gene (*tzs*) was kindly provided by Dr. Jessica Erickson (IPB, Halle) (Erickson et al., 2014).

*Infiltration of N. benthamiana leaves*

Different *Agrobacterium* strains were grown on LB agar medium containing the respective antibiotics for two days and resuspended in *Agrobacterium* infiltration medium (AIM: 10 mM MES, 10 mM $MgCl_2$, 10 µM acetosyringone). Cultures were adjusted to $OD_{600}$ of 0.2 and incubated for two hours at room temperature. Cultures were then infiltrated into leaves of five-week-old *Nicotiana benthamiana* plants. For cytokinin infiltration, each phytohormone (*trans*-Zeatin, kinetin, and 6-benzylaminopurine (BAP), from Duchefa Biochemie B.V. Haarlem, The Netherlands) was first dissolved in 0.5 mL of 1 N NaOH and subsequently in water for 100 µg $ml^{-1}$ working solutions. Plants were infiltrated three times, every other day. Multiple CK infiltrations were required to produce clear effects, suggesting that continuous CK supply—as provided by ongoing bacterial *tzs* expression—is key for full trichome morphogenesis.

The vector pAGM50583 for betalain biosynthesis (Grützner et al., 2021) was used to help visualize the trichomes, which appear white on a red background.

*Trichome counting and imaging*

Leaf discs were excised from infiltrated spots using a cork borer (diameter 1 cm). Trichomes were manually counted on 1-cm diameter leaf discs with the VHX-6000 microscope in combination with a VH-Z20 T zoom lens (Keyence, Osaka, Japan). For trichome counting on adaxial and abaxial sides at different leaf developmental stages, leaf discs of infiltrated areas were dipped in a 0.1% w/v Rhodamine B (Sigma) solution for 5 min. Images of the fluorescent trichomes were acquired using a Zeiss Axio Zoom.V16 microscope (excitation 559-585; emission 600-690 nm). Images shown in Figure 1B, 1F were obtained by cutting 1.5 mm wide leaf strips and rotating them 90°. Environmental scanning electron microscopy images were recorded using an ESEM XL-30 FEG (FEI/Philips, Eindhoven, The Netherlands) at specified time points after agroinfiltration.

*Scanning electron microscopy*

Leaf samples were excised into approximately 5 × 5 mm pieces near the infiltration site and immediately fixed in 3% glutaraldehyde prepared in 0.1 M sodium cacodylate buffer (pH 7.2) for 2 h. Following fixation, samples were rinsed in 0.1 M sodium cacodylate buffer for 10 min and dehydrated through a graded ethanol series (30%, 50%, 70%, 90%, 95%, and 100%). For critical point drying (CPD), samples were incubated in an ethanol/acetone mixture (1:1, v/v) for 45 min, followed by 100% acetone for at least 2 h prior to drying using a critical point dryer. Dried samples were mounted onto SEM stubs using double-sided adhesive tape.
Scanning electron microscopy (SEM) imaging was performed using a Zeiss Gemini 500 at an accelerating voltage of 1 kV using secondary electron (SE) detection. Images were acquired at stage tilt angles of 0° and 40–45°.

*Acylsugar profiling*

Leaf surface extracts were obtained by dipping one 1-cm-diameter leaf disc into 0.5 mL of 80% methanol solution and inverting the tubes for 2 min. Representative acylsugars (AS) produced by *Nicotiana benthamiana* glandular trichomes were quantified by LC-MS/MS. Metabolites were separated with an Acquity UPLC (Waters Inc.) with a Nucleoshell RP18 column (Macherey & Nagel, 150 mm × 2 mm × 2.7 µm). Solvents and gradient were adjusted according to Balcke et al. (2024). Mass spectrometric analysis was performed by MS1 full scan from 65-1500 Daltons and 100 ms accumulation time (ZenoToF 7600, Sciex GmbH, Darmstadt, Germany) operating in negative mode and controlled by Sciex OS software (Sciex). The identity of specific AS was confirmed by MS/MS spectral matching to an inhouse library. MS1 AS peak areas were determined with Multiquant 3 software (Sciex GmbH, Darmstadt, Germany).

*Cytokinin measurements*

Cytokinin quantification was performed using 50 mg of homogenized plant tissue extracted with 400 µl of 80% (v/v) methanol containing 25 pg.µl$^{-1}$ *trans*-zeatin-[d5] and 50 pg.µl$^{-1}$ *trans*-zeatin-O-glucoside-[d5] as stable isotope-labeled internal standards. Extracts were centrifuged twice, and the supernatants were collected for analysis. A 10 µl aliquot was injected into an ACQUITY UPLC system (Waters Inc.) equipped with a Nucleodur Gravity C18 SB column (1.8µm EC 100/2 Macherey Nagel, Dueren, Germany). The mobile phase consisted of solvent A (0.1% (v/v) aqueous formic acid) and solvent B (acetonitrile / 0.1% (v/v) formic acid). The gradient was as follows: 98% A for 2 minutes; a linear gradient from 98% to 5% A from 2 to 14 minutes; isocratic 5% A from 14 to 17.5 minutes; isocratic 98% A from 18 to 20 minutes. The flow rate was 0.5 ml min$^{-1}$, and the column temperature was maintained at 40°C. Analytes were detected using a Sciex TripleQuad 7500+ mass spectrometer (AB Sciex, Darmstadt, Germany.operating in positive electrospray

ionization mode with scheduled multiple reaction monitoring (MRM). Peaks were automatically integrated by the MQ4 algorithm embedded in the Sciex OS 4.0 software (AB Sciex, Darmstadt, Germany), and integration was manually adjusted if necessary. Quantification of trans-zeatin and trans-zeatin-O-glucoside was performed based on the ratio of analyte to corresponding internal standard peak areas.

*RNA extraction and sequencing*

Total RNA was extracted from *N. benthamiana* leaf discs infiltrated with the different *Agrobacterium* strains, using the RNeasy© Plant Mini Kit (Qiagen United States, 74904) and following the manufacturer's instructions. RNA integrity (RIN > 7.0) was evaluated with BioAnalyzer utilizing the RNA 6000 Nano kit (Agilent Technologies). Library preparation and mRNA sequencing was conducted by the company Novogen UK (www.novogene.com), using Illumina NovaSeq 6000 Sequencing PE150, Q30 ≥85%. Reads were mapped to the *N. benthamiana* genome (Nbe_v1.1) (Kurotani et al., 2023) using the nf-core/rnaseq pipeline (Ewels et al., 2020) with the default parameters. Differential expression analysis was performed using DESeq2 (Love et al., 2014). PCA and heatmap were plotted using the SRplot website (Tang et al., 2023). Venn diagram was constructed using InteractiVenn (Heberle et al., 2015). GO term enrichment analysis was conducted in RStudio 2026.01.0 using ClusterProfiler package. Raw sequencing reads have been deposited in the NCBI Bioproject accession PRJNA1424681.

*Statistical analysis*

Statistical analyses were performed using GraphPad Prism 9.5.1 software (https://www.graphpad.com/). Pairwise comparisons were conducted using Student's *t*-test with a significance threshold of $p < 0.05$. For multiple group comparisons, one-way ANOVA was used followed by Tukey's post hoc test, with $p < 0.05$ considered significant.

## References


**Balcke, G.U., Vahabi, K., Giese, J., Finkemeier, I., and Tissier, A.** (2024). Coordinated metabolic adaptation of Arabidopsis thaliana to high light. The Plant Journal **120**:387-405. https://doi.org/10.1111/tpj.16992.

**Erickson, J.L., Ziegler, J., Guevara, D., Abel, S., Klösgen, R.B., Mathur, J., Rothstein, S.J., and Schattat, M.H.** (2014). Agrobacterium-derived cytokinin influences plastid morphology and starch accumulation in Nicotiana benthamiana during transient assays. BMC Plant Biology **14**:127. 10.1186/1471-2229-14-127.

**Ewels, P.A., Peltzer, A., Fillinger, S., Patel, H., Alneberg, J., Wilm, A., Garcia, M.U., Di Tommaso, P., and Nahnsen, S.** (2020). The nf-core framework for community-curated bioinformatics pipelines. Nature Biotechnology **38**:276-278. 10.1038/s41587-020-0439-x.

**Grützner, R., Schubert, R., Horn, C., Yang, C., Vogt, T., and Marillonnet, S.** (2021). Engineering Betalain Biosynthesis in Tomato for High Level Betanin Production in Fruits. Frontiers in Plant Science **12**:682443.

**Heberle, H., Meirelles, G.V., da Silva, F.R., Telles, G.P., and Minghim, R.** (2015). InteractiVenn: a web-based tool for the analysis of sets through Venn diagrams. BMC Bioinformatics **16**:169. 10.1186/s12859-015-0611-3.

**Hynes, M.F., Simon, R., and Pühler, A.** (1985). The development of plasmid-free strains of Agrobacterium tumefaciens by using incompatibility with a Rhizobium meliloti plasmid to eliminate pAtc58. Plasmid **13**:99-105. https://doi.org/10.1016/0147-619X(85)90062-9.

**Kurotani, K.-i., Hirakawa, H., Shirasawa, K., Tanizawa, Y., Nakamura, Y., Isobe, S., and Notaguchi, M.** (2023). Genome Sequence and Analysis of Nicotiana benthamiana, the Model Plant for Interactions between Organisms. Plant and Cell Physiology **64**:248-257. 10.1093/pcp/pcac168.

**Love, M.I., Huber, W., and Anders, S.** (2014). Moderated estimation of fold change and dispersion for RNA-seq data with DESeq2. Genome Biology **15**:550. 10.1186/s13059-014-0550-8.

**Tang, D., Chen, M., Huang, X., Zhang, G., Zeng, L., Zhang, G., Wu, S., and Wang, Y.** (2023). SRplot: A free online platform for data visualization and graphing. PLoS One **18**:e0294236. 10.1371/journal.pone.0294236.
